# Therapeutic impact of basic critical care echocardiography performed by residents after limited training

**DOI:** 10.1186/s13613-024-01354-7

**Published:** 2024-07-29

**Authors:** Marine Goudelin, Bruno Evrard, Roxana Donisanu, Céline Gonzalez, Christophe Truffy, Marie Orabona, Antoine Galy, François-Xavier Lapébie, Yvan Jamilloux, Elodie Vandeix, Dominique Belcour, Charles Hodler, Lucie Ramirez, Rémi Gagnoud, Catherine Chapellas, Philippe Vignon

**Affiliations:** 1 Medical-Surgical Intensive Care Unit, Dupuytren University Hospital, 87000 Limoges, France; 2 Inserm CIC1435, 87000 Limoges, France

**Keywords:** Transthoracic echocardiography, Training, Intensive care unit, Point of care technology, Therapeutic uses, Ultrasound

## Abstract

**Background:**

The objective was to assess the agreement between therapeutic proposals derived from basic critical care echocardiography performed by novice operators in ultrasonography after a limited training (residents) and by experts considered as reference. Secondary objectives were to assess the agreement between operators’ answers to simple clinical questions and the concordance between basic two-dimensional measurements.

**Methods:**

This observational, prospective, single-center study was conducted over a 3-year period in a medical-surgical intensive care unit. Adult patients with acute circulatory and/or respiratory failure requiring a transthoracic echocardiography (TTE) examination were studied. In each patient, a TTE was performed by a resident novice in ultrasonography after a short training program and by an expert, independently but within 1 h and in random order. Each operator addressed standardized simple clinical questions and subsequently proposed a therapeutic strategy based on a predefined algorithm.

**Results:**

Residents performed an average of 33 TTE studies in 244 patients (156 men; age: 63 years [52–74]; SAPS2: 45 [34–59]; 182 (75%) mechanically ventilated). Agreement between the therapeutic proposals of residents and experienced operators was good-to-excellent. The concordance was excellent for suggesting fluid loading, inotrope or vasopressor support (all Kappa values > 0.80). Inter-observer agreement was only moderate when considering the indication of negative fluid balance (Kappa: 0.65; 95% CI 0.50–0.80), since residents proposed diuretics in 23 patients (9.5%) while their counterparts had the same suggestion in 35 patients (14.4%). Overall agreement of responses to simple clinical questions was also good-to-excellent. Intraclass correlation coefficient exceeded 0.75 for measurement of ventricular and inferior vena cava size.

**Conclusions:**

A limited training program aiming at acquiring the basic level in critical care echocardiography enables ICU residents novice in ultrasonography to propose therapeutic interventions with a good-to-excellent agreement with experienced operators.

**Supplementary Information:**

The online version contains supplementary material available at 10.1186/s13613-024-01354-7.

## Background

Critical care echocardiography (CCE) is performed and interpreted by the frontline intensivist at the bedside for diagnostic purpose and to best guide the management of ICU patients with acute circulatory and/or respiratory failure [1, 2]. Basic CCE mainly refers to the use of transthoracic echocardiography (TTE) to perform a targeted qualitative assessment to answer few simple clinical questions [1]. It should be mastered by all intensivists at the end of their initial training [3, 4]. A limited training enables non-cardiologist ICU residents without previous experience in ultrasonography to accurately answer simple clinical questions based on basic CCE [5–9]. Nevertheless, studies evaluating the efficiency of training programs to reach proficiency in basic CCE are scarce and the ability of trainees to provide adequate therapeutic proposals derived from basic CCE has not yet specifically been evaluated.

Accordingly, we sought to assess the reliability of therapeutic proposals directly derived from basic CCE when performed by non-cardiologist residents without previous experience in ultrasonography and after a limited training, in ICU patients with cardiopulmonary compromise, when using experienced operators as reference.

## Patients and methods

### Study design

This observational, prospective and single-center study was conducted during a 3-year period in the medical-surgical ICU of a French Teaching Hospital. ICU residents participated in the study on a voluntary basis if they had no previous experience in self-performed echocardiography examination. Experience in ultrasound-guided catheterization was accepted for residents in Anesthesiology. Patients were assessed by both the residents recently trained to basic CCE and an intensivist with expertise in CCE who was considered the reference. Focused TTE examinations were performed randomly according to the availability of operators, and independently but within 1 h. No therapeutic intervention was performed between both TTE assessments or based on the resident’s assessment. The study was approved by the local Ethics Committee which waived the need for informed consent due to its observational nature.

### Study population

Consecutive patients over 18 years were eligible when they needed hemodynamic assessment using TTE for an acute circulatory and/or respiratory failure. In each patient, the reason for ICU admission, the Simplified Acute Physiologic Score (SAPS) II, ventilator settings, vital parameters, biology and ongoing treatment at the time of TTE assessment were recorded.

### Training program

Fifteen residents (Anesthesiology: n = 9; Nephrology: n = 3; Internal medicine: n = 2; Pneumology: n = 1) underwent a 4-h theoretical training program as part of a previously validated curriculum [6]. This theoretical training was followed by 2 h of illustrative clinical cases and 6 h of tutored hands-on training at patients’ bedside to apply the acquired theoretical knowledge. Hands-on sessions were initially performed on 10 to 12 patients to master the technical aspects of TTE examination, images acquisition and adequate identification of anatomical structures. Four to 6 additional TTE examinations allowed residents to adequately complete the case report form and to use the proposed algorithm. The training program was conducted by experienced intensivists with expertise in CCE [1]. The 6-month rotation of participating residents was typically organized as follows: 1 month to become familiar with ICU standards of care and organization, 1 month of basic CCE training, and participation in the study during the 4 remaining months.

### Point-of-care echocardiography

TTE examinations were performed using a full-feature compact system CX 50 (Philips Healthcare) with a 3.5–5 MHz probe. The parasternal long- and short-axis views, the apical-four chamber view, the subcostal and inferior vena cava (IVC) views were systematically screened in all patients. Quality of two-dimensional images was assessed in each TTE view as follows: 0, no image; 1, poor quality (identification of less than 50% of the endocardium of the ventricles or of the IVC); 2, good quality (identification of more than 50% of the endocardium of the ventricles or of the IVC); 3, excellent quality (complete identification of the endocardium of the ventricles or of the IVC) [5]. Measurements were performed on-line on two-dimensional freeze frames at end-expiration: left ventricular (LV) end-diastolic diameter in the parasternal long-axis view, right ventricular (RV) and LV end-diastolic diameter ratio (RVEDD/LVEDD) in the apical-four chamber view, and maximal IVC diameter [6].

In each patient, the operators answered simple clinical questions to cover the field of competence of basic CCE [1]: global LV systolic function based on the visual assessment of LV ejection fraction (EF), homogeneity or heterogeneity (i.e., abnormal wall motion abnormality) of LV contraction, LV cavity size, RV size and systolic function, presence of paradoxical septal motion, pericardial effusion and potential tamponade, IVC size and respiratory variations (in spontaneously breathing patients), and severe mitral or aortic insufficiency identified using color Doppler mapping [10]. Global LV systolic dysfunction was considered moderate for a visually estimated LVEF between 30 and 50%, and severe when LVEF was lower than 30% [6]. RV systolic function was assessed visually in the apical four-chamber view, and if not available, in the subcostal long-axis view. Visual assessment of the systolic tilt of the tricuspid annulus (lateral aspect) and thickening of RV free wall were qualitatively evaluated as normal or depressed. LV end-diastolic diameter > 52 mm for women and > 58 mm for men defined LV dilatation [11]. RV dilatation corresponded to a RVEDD/LVEDD ratio > 0.6 [12]. Acute cor pulmonale was defined by a RV dilatation associated with a paradoxical septal motion in the parasternal short-axis view [13]. IVC dilatation was defined by an end-expiratory diameter > 23 mm [14].

Residents and experienced operators had the same access to clinical information and available results of laboratory tests and other imaging techniques. Residents never assisted to an echocardiography examination performed in the same patients before their evaluated TTE. During the evaluation period, each patient was only assessed once and by a single resident. They independently performed and interpreted CCE examination with 11 simple clinical questions to systematically address, and then filled out a dedicated clinical research form on-line. To avoid any confusion bias, the experienced operator filled his clinical research form before completing the full comprehensive CCE assessment (e.g., use of advanced indices or transesophageal echocardiography), when indicated for patient management.

Each operator proposed a therapeutic strategy based on a predefined algorithm (Table 1). In patients sustaining a circulatory failure, a hyperkinetic LV with small cardiac cavities and an IVC diameter < 12 mm with marked inspiratory collapse in spontaneously breathing patients were consistent with overt hypovolemia requiring fluid loading. LV systolic dysfunction without concomitant signs of hypovolemia but associated dilatation of the left atrium was indicative of congestive heart failure and inotropes were then proposed according to its severity. In a patient with acute respiratory failure, a dilatation of the left atrium associated with a constant bulging of the interatrial septum towards the right atrium indicated elevated LV filling pressure and suggested a potential cardiogenic pulmonary edema with indication for diuretic therapy. Vasopressor support, protective ventilation, prone ventilation and inhaled nitric oxide were suggested in the presence of RV dysfunction and/or acute cor pulmonale, when associated with systemic venous congestion (dilated IVC without inspiratory collapse in spontaneously breathing patients). Associated dilatation of the right atrium and constant bulging of the interatrial septum towards the left atrium indicated elevated RV filling pressure. Inotropes could be proposed in the presence of severe biventricular systolic dysfunction. In hypotensive patients, the presence of a normal or increased global systolic function of both ventricles without signs of hypovolemia or other relevant abnormalities (e.g., tamponade, acute valvular disease) was consistent with sustained vasoplegia, especially when LV cavity was virtual at end-systole, and led to propose a vasopressor support. Diagnosis of tamponade or acute and severe left-sided valvular regurgitation led to propose pericardiocentesis or prompt valvular surgery, respectively (Table 1).

**Table 1:** Therapeutic algorithm according to the results of basic CCE performed for acute circulatory and/or respiratory failure

Echocardiographic parameters and derived therapeutic proposals	Hypovolemia	LV systolic dysfunction	RV dysfunction (± acute cor pulmonale)	Tamponade	Acute massive MR or AR	Vasoplegia
LV systolic function	N or ↑	↓ to ↓↓	N	N	N to ↑	↑ to ↑↑
LV size	↓ to ↓↓	N or ↑ to ↑↑^a^	N or ↓	N or ↓	N	N or ↓ (± end-systolic obliteration)
RV size	↓ to ↓↓	N	↑ to ↑↑^b^ (± paradoxical septal motion)	↓ to ↓↓	N	N
Pericardial effusion	–	–	–	+ to +++	–	–
Maximal IVC diameter/respiratory variation of IVC size in spontaneously breathing patients	< 12 mm/total inspiratory collapse	–	> 23 mm/little or no respiratory variation	> 23 mm/no respiratory variation	–	–
Color Doppler mapping	–	–	–	–	Massive regurgitation	–
Therapeutic proposals	Fluid loading	Inotropes^c^	Vasopressor^d^ ± protective ventilation ± prone ventilation ± inhaled nitric oxide	Pericardiocentesis	Emergency valve surgery	Vasopressor support

*CCE* critical care echocardiography, *LV* left ventricle, *RV* right ventricle, *IVC* inferior vena cava, *N* normal, *MR* mitral regurgitation, *AR* aortic regurgitation

^a^A visually dilated left atrium together with a constant bulging of the interatrial septum towards the right atrium throughout the cardiac and respiratory cycle was consistent with elevated left ventricular filling pressures

^b^A visually dilated right atrium together with a constant bulging of the interatrial septum towards the left atrium throughout the cardiac and respiratory cycle was consistent with elevated right ventricular filling pressures, hence systemic venous congestion

^c^Diuretics could be proposed to reduce fluid balance in hemodynamically stable patients with respiratory compromise

^d^Inotropes may be discussed in the presence of severe biventricular systolic dysfunction

### Statistical analysis

Results are expressed as medians and interquartiles, or as percentages. Differences between the groups were testing using Mann–Whitney U test and Person’s Chi-squared test. The agreement of therapeutic proposals and answers to clinical questions between trainees and experts used as reference was assessed using the Cohen’s Kappa coefficient and its 95% confidence intervals (CI) [15]. Concordance of measurements performed by trainees and experts was assessed using the intraclass correlation coefficient and its 95% CIs. A *p* value < 0.05 was considered statistically significant. All data were generated using R software (4.2.1).

## Results

Among 265 eligible patients, 21 patients (8%) could not be evaluated due to the absence of TTE images, even when assessed by the experienced operator. Accordingly, 244 patients were studied (156 men; age: 63 years [52–74]; SAPS2: 45 [34–59], 182 of them (75%) being mechanically ventilated). Indications for TTE assessment were mainly the presence of an acute circulatory or respiratory failure (either isolated or combined), a resuscitated cardiac arrest, or patients difficult to wean from the ventilator (Table 2). Residents performed an average of 33 ± 4 TTE studies (range: 29–39), including 16 ± 4 TTE examinations during the evaluation period (n = 244). Experienced operators performed the examinations faster than the residents (4 min [3–5] vs. 12 min [10–17]: p < 0.001). The proportion of good-to-excellent image quality was significantly higher with the experienced operators [1000/1220 views (82%) vs. 903/1220 views (74%): p < 0.0001]. Image quality for each TTE view and the proportion of patients in whom each TTE parameter could have been obtained by investigators are indicated in the Supplementary Table 1.

**Table 2:** Characteristics of the study population

Baseline characteristics (n = 244)	
Age (years)	63 (52–74)
Gender, male, n (%)	156 (64)
BMI (kg/m^2^)	26 (24–28)
SAPS 2 score	45 (34–59)
Invasive mechanical ventilation, n (%)	182 (75)
Tidal volume (ml/kg)	7.0 (6.3–7.7)
Positive end-expiratory pressure (cmH_2_O)	6 (6–8)
Heart rate (bpm)	99 (85–115)
Mean blood pressure (mmHg)	84 (73–95)
Sinus rhythm, n (%)	194 (80)
Vasopressor, n (%)	134 (55)
Dose of vasopressor^a^	
Norepinephrine (μg/kg/min)	1.47 (0.74–3.28) (50^b^)
Epinephrine (μg/kg/min)	1.65 (0.50–3.19) (26^b^)
Reason for ICU admission, n (%)	
Medical	233 (95.5)
Surgical	11 (4.5)
Indication for basic critical care echocardiography, n (%)	
Acute circulatory failure	91 (37)
Acute respiratory failure	80 (33)
Combined circulatory and respiratory failure	20 (8)
Cardiac arrest	48 (20)
Difficult weaning from the ventilator	5 (2)

*SAPS* simplified acute physiology score, *ICU* intensive care unit

^a^A single patient could receive several catecholamines

^b^Numbers between brackets indicate percentages

**Fig. 1:**
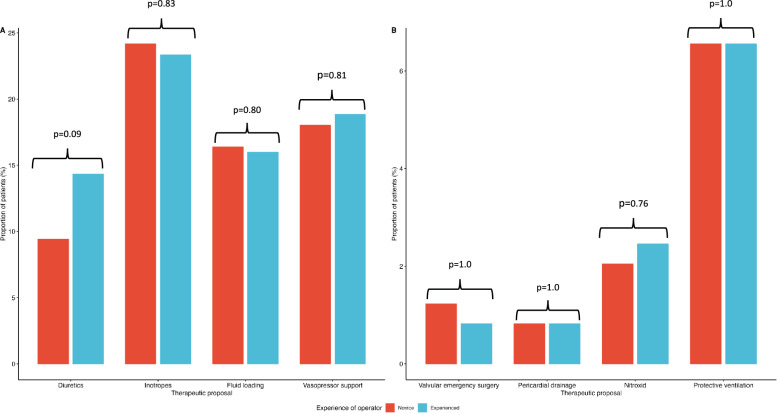
Agreement between the therapeutic proposals of novice operators (residents) and experienced operators

The proportion of therapeutic proposals was not significantly different between recently trained residents and experienced operators [153/244 (63%) vs. 165/244 (68%); p = 0.3]. Agreement between the therapeutic proposals of residents and experienced operators was good-to-excellent (Table 3, Fig. 1). Inter-observer concordance was excellent for suggesting fluid loading, inotrope or vasopressor support, or less frequently to propose a more protective ventilation and the administration of inhaled nitric oxide (all Kappa values > 0.80). Residents accurately identified the two indications of pericardiocentesis (Table 3). In contrast, they proposed discussing a prompt surgical repair in a patient with a severe mitral insufficiency which did not meet the criteria for an emergency correction. Inter-observer agreement was only moderate when considering the indication of negative fluid balance (Kappa: 0.65; 95% CI 0.50–0.80), since residents proposed diuretics in 23 patients (9.5%) while their counterparts had the same suggestion in 35 patients (14.4%) (Table 3).

**Table 3:** Therapeutic proposals emanating from residents and experienced operators based on the interpretation of basic critical care echocardiography (244 examinations)

Therapeutic proposals	Novice operator	Experienced operators	*p*	K concordance coefficient^a^
Fluid loading, n (%)	40 (16)	39 (16)	0.80	0.82 (0.72–0.92)
Vasopressor support, n (%)	44 (18)	46 (19)	0.81	0.86 (0.78–0.95)
Inotrope, n (%)	59 (24)	57 (23)	0.83	0.82 (0.73–0.90)
Diuretics, n (%)	23 (9)	35 (14)	0.09	0.65 (0.50–0.80)
Protective ventilation, n (%)	16 (7)	16 (7)	1.0	1 (1–1)
Inhaled nitric oxide, n (%)	5 (2)	6 (2.5)	0.76	0.91 (0.66–1)
Pericardiocentesis, n (%)	2 (0.8)	2 (0.8)	1.0	1 (1–1)
Emergency valve surgery, (%)	3 (1)	2 (0.8)	1.0	0.80 (0.41–1)

^a^95% CIs are noted in parentheses

The number of unanswered clinical questions was lower with the experienced operators than with residents [406/2684 (15%) vs. 485/2684 (18%); p = 0.004]. Pericardial effusions and IVC dilatation were more frequently diagnosed by experts [236/244 (97%) vs. 226/244 (93%); p = 0.04; 209/244 (86%) vs. 192/244 (79%); p = 0.04, respectively]. When clinical questions were addressed by both the residents and experienced operators, the overall agreement was good-to-excellent (Supplementary Table 2). The concordance between measurements of LV end-diastolic diameter and RV/LV end-diastolic diameter ratio performed by both operators was excellent (intraclass correlation coefficient: 0.83, 95% CI 0.77–0.87; 0.81, 95% CI 0.76–0.86, respectively) and the concordance for IVC maximal diameter was good (0.78, 95% CI 0.62–0.86). Mean bias for measurement of LV end-diastolic diameter, RV/LV end-diastolic diameter and IVC maximal diameter between residents and experts was − 1.46 mm, − 0.02 and − 1.54 mm, respectively, with fairly large standard deviations (Supplementary Fig. 1).

## Discussion

This is the first study showing that therapeutic proposals derived from bedside interpretation of basic CCE by residents, without previous experience in ultrasound and after a limited training, were concordant with those suggested by experienced operators with the same level of information. This was related to a good-to-excellent agreement between answers to clinical questions covering the field of competence of basic CCE provided by novices and experts [1], and to the concordance of simple two-dimensional measurements performed by investigators.

Therapeutic proposals emanating from residents with basic level in CCE had a good-to-excellent agreement with those of the experienced intensivists. The agreement was excellent for fluid loading, vasopressor support, inotropes, protective ventilation, inhaled nitric oxide and pericardiocentesis. Jozwiak et al. [7] previously showed in ventilated patients assessed using TTE for circulatory failure that the agreement regarding both the final diagnosis and therapeutic proposal between residents novice in ultrasound after a dedicated training and experts improved during the 6-month rotation. Nevertheless, they validated prospectively a skills assessment score which did neither include quantitative measurements nor therapeutic proposals [7]. In our study, inter-operator agreement was lower for diuretic therapy to reduce fluid balance in patients with respiratory compromise (e.g., acute respiratory failure, difficult ventilator weaning). Reasons for the underestimation of indication for diuretic therapy by recently trained residents are threefold. First, the decision to reduce fluid balance mainly relies on the interpretation of both the clinical context and patient status, which requires medical experience. Second, basic CCE findings reflecting underlying elevation of LV filling pressures are indirect, frequently subtle, and require experience for accurate identification and interpretation (e.g., cardiomyopathy known to be associated with LV diastolic dysfunction, left-sided valvular regurgitation associated with left atrial dilatation, constant right bulging of interatrial septum). Third, even when using advanced CCE with all Doppler capabilities to assess LV diastolic properties and filling pressure [16], accurate diagnosis of pulmonary venous congestion remains at times challenging in experienced hands [17]. In this specific clinical setting, combined lung ultrasound has been shown to be complementary to CCE [18, 19].

In keeping with our previous studies [5, 6], residents adequately assessed both the systolic function and size of the two ventricles, the presence of a pericardial effusion and IVC diameter as a marker of preload and potential systemic venous congestion. They disagreed more frequently with the experienced operators on the presence or not of an inspiratory collapse of the IVC in spontaneously breathing patients. IVC diameter could not be measured in 52/244 patients (21%) by the residents and in 35/244 patients (14%) by the experienced operator, which is in keeping with the 22% reported in a recent study due to poor image quality in the subcostal view [20]. In our study, pericardial effusions were accurately identified by the recently trained residents, as well as the two cases of tamponade. Similarly, residents accurately identified acute severe left-sided regurgitation. In one patient, the severity of mitral regurgitation was overestimated. This confirms that the indication for prompt valvular surgery must be discussed with an experienced echocardiographer [1, 6]. In the present study, overall good-to-excellent diagnostic performance of recently trained residents partially relied on the performance of 33 TTE during their 6-month rotation on average, a number of examinations similar to that reported previously [5, 6], which takes into account the learning curve [21]. These results substantiate current recommendations suggesting the performance of at least 30 examinations to reach competence in basic CCE [4, 9].

Targeted ultrasound examination performed by the frontline intensivist has become a simple and efficient tool to improve information yielded by the sole clinical examination in unstable ICU patients [2, 22]. Point-of-care ultrasonography reduces costs in accelerating diagnostic process and therapeutic decisions [23, 24]. Although in unstable patients typical CCE abnormalities are frequently observed, hence easier to identify and interpret, an intensivist novice in ultrasound may inadequately scan the patient or erroneously interpret CCE findings. Such diagnostic error may have potentially serious consequences and legal issues [25]. Although erroneous diagnoses (false positive results) or missed diagnoses (false negative results) may lead to inadequate therapy with dramatic consequences in ICU patients, only few medico-legal issues have yet been reported [26]. Nevertheless, with the increasing use of point-of-care ultrasound and accumulated validation of focused training program to reach competence in basic CCE, one may anticipate a legal risk for physicians for *not* using this diagnostic approach when indicated [27]. The use of basic CCE by the attending physician in the specific clinical context of a given patient as an adjunct to clinical examination rather than instead the conventional diagnostic work-up appears the best strategy to minimize such risks [28]. In patients with inadequate surface image quality, TEE has been shown to be feasible and safe in ICU ventilated patients when performed by novices after a limited training [8, 29, 30]. Artificial intelligence promises to help guiding TTE image acquisition and interpretation by novices, as suggested by preliminary studies performed in Cardiology [31]. Nevertheless, there are still no strong guidelines for training or competence process required for basic point-of-care echocardiography in intensive care [3, 32–34].

Our study has several limitations. The relevance of therapeutic proposals emanating from the experienced operators after basic CCE has not been assessed in terms of efficiency and tolerance. To avoid confusion bias, we purposely did not assess the additional value of completing the basic examination performed by experienced operators by an advanced assessment, including TEE when needed. The standardized therapeutic algorithm relied on non-sensitive findings to be in accordance with basic level CCE [1]. Although we only evaluated a rather small sample of novice operators, residents had various medical background and are representative of main medical specialties. The case-mix presented in the present cohort may not be representative of other ICUs. Moreover, the additional value of CCE assessment when compared to the sole clinical approach has not been evaluated. Finally, chest ultrasound which can also be performed by novices after a limited training, especially in patients with acute respiratory failure [35–37], was not used as an adjunct of basic CCE assessment in patients examined for acute respiratory failure.

## Conclusion

In ICU patients with hemodynamic and/or respiratory failure, a limited training program aiming at reaching basic level in CCE enables residents novice in ultrasonography to propose acute therapy with a good-to-excellent agreement with the experienced operators. This study also confirms that the proposed curriculum and the performance of at least 30 examinations enable ICU residents to adequately answer to simple clinical questions covering the field of knowledge of basic CCE, with a good agreement with experienced operators. Whether routine use of basic CCE improves outcome of ICU patient remains to be determined.

### Supplementary Information

Supplementary Material 1: Figure S1. Agreement between two-dimensional measurements performed by novice operatorsand experienced operators according to the Bland and Altman representation. Mean biases are indicated by the red dotted line and 1.96 standard deviations by the green dotted lines for the measurement of left ventricular end-diastolic diameter in the parasternal long-axis view, the ratio of right ventricular and left ventricular end-diastolic diameter measured in the apical four-chamber view, and for the end-expiration diameter of the inferior vena cava. Abbreviations: LVEDD, left ventricular end-diastolic diameter; RVEDD, right ventricular end-diastolic diameter; IVC, inferior vena cava; EO, experience operator; NO, novice operator; SD, standard deviation.

Supplementary Material 2.

Supplementary Material 3.

## Data Availability

The datasets used and/or analysed during the current study are available from the corresponding author on reasonable request.

## Abbreviations

CCE: Critical care echocardiography
CI: Confidence interval
EDD: End-diastolic diameter
EF: Ejection fraction
ICU: Intensive care unit
IVC: Inferior vena cava
LV: Left ventricle
RV: Right ventricle
SAPS: Simplified acute physiology score
TTE: Transthoracic echocardiograph
