# Supplementary figures and images for: Therapeutic impact of basic critical care echocardiography performed by residents after limited training
Source: Ann Intensive Care. 2024 Jul 29;14:119. doi: 10.1186/s13613-024-01354-7 (PMC11286607; doi:10.1186/s13613-024-01354-7)

A

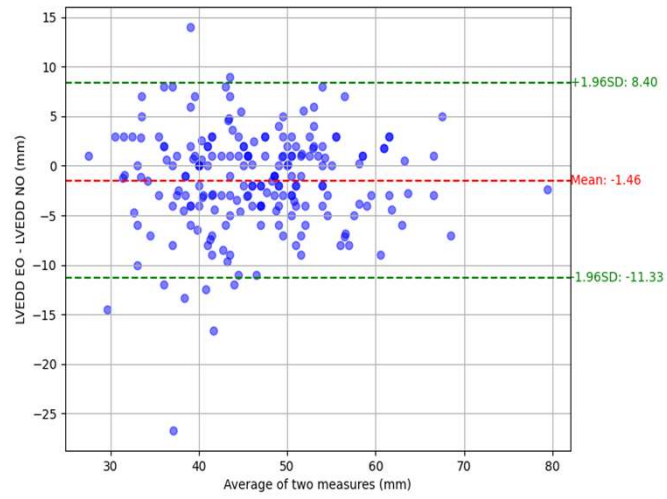

B

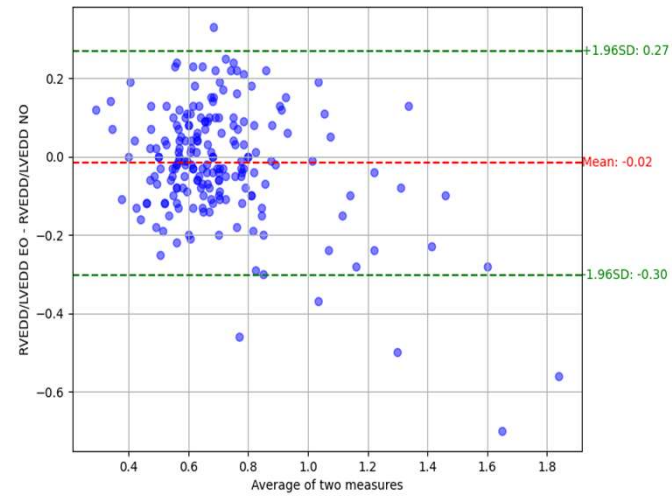

C

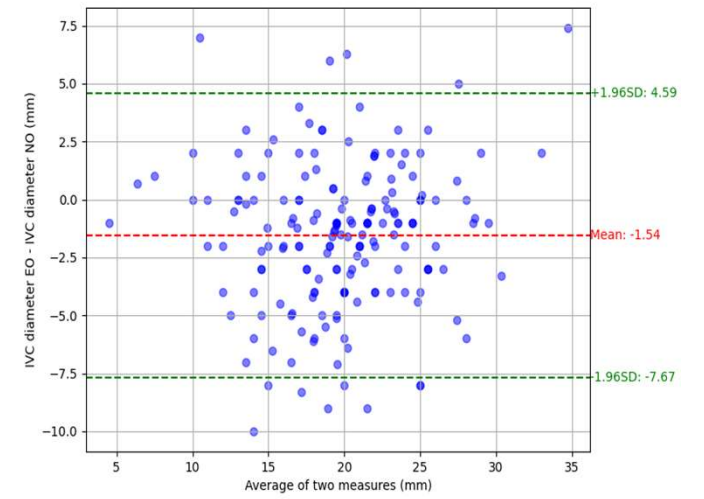

Supplement: Supplementary file 1 — Supplementary Material 1: Figure S1. Agreement between two-dimensional measurements performed by novice operatorsand experienced operators according to the Bland and Altman representation. Mean biases are indicated by the red dotted line and 1.96 standard deviations by the green dotted lines for the measurement of left ventricular end-diastolic diameter in the parasternal long-axis view, the ratio of right ventricular and left ventricular end-diastolic diameter measured in the apical four-chamber view, and for the end-expiration diameter of the inferior vena cava. Abbreviations: LVEDD, left ventricular end-diastolic diameter; RVEDD, right ventricular end-diastolic diameter; IVC, inferior vena cava; EO, experience operator; NO, novice operator; SD, standard deviation. [file 13613_2024_1354_MOESM1_ESM.pdf]
