# Supplementary material for: Therapeutic impact of basic critical care echocardiography performed by residents after limited training
Source: Ann Intensive Care. 2024 Jul 29;14:119. doi: 10.1186/s13613-024-01354-7 (PMC11286607; doi:10.1186/s13613-024-01354-7)
Supplement: Supplementary file 2 — Supplementary Material 2. [file 13613_2024_1354_MOESM2_ESM.doc]

**Supplementary Table 1:** Image quality and missing measurements provided by residents and experienced operators during basic critical care echocardiography (244 examinations)

| |  | Residents | Experienced operators | *p* | | --- | --- | --- | --- | | **Image quality** | | | | | ***Parasternal long-axis view:*** |  |  | 0.1 | | Excellent, n (%) | 107 (44) | 134 (55) |  | | Good, n (%) | 83 (34) | 72 (30) |  | | Poor, n (%) | 38 (16) | 28 (11) |  | | No image, n (%) | 15 (6.2) | 10 (4.1) |  | | Unknown, n (%) | 1 (0.004) | 0 (0) |  | | ***Parasternal short-axis view:*** |  |  | 0.014 | | Excellent, n (%) | 90 (37) | 124 (51) |  | | Good, n (%) | 81 (33) | 71 (29) |  | | Poor, n (%) | 42 (17) | 27 (11) |  | | No image, n (%) | 30 (12) | 22 (9) |  | | Unknown, n (%) | 1 (0.004) | 0 (0) |  | | ***Apical- four chamber view:*** |  |  | < 0.001 | | Excellent, n (%) | 100 (24) | 155 (64) |  | | Good, n (%) | 104 (43) | 57 (23) |  | | Poor, n (%) | 25 (10) | 26 (11) |  | | No image, n (%) | 14 (5.8) | 6 (2.5) |  | | Unknown, n (%) | 1 (0.004) | 0 (0) |  | | ***Subcostal view:*** |  |  | < 0.001 | | Excellent, n (%) | 74 (30) | 128 (52) |  | | Good, n (%) | 77 (32) | 51 (21) |  | | Poor, n (%) | 18 (7.4) | 15 (6.1) |  | | No image, n (%) | 48 (20) | 27 (11) |  | | Unknown, n (%) | 1 (0.004) | 0 (0) |  | | ***IVC view:*** |  |  | < 0.001 | | Excellent, n (%) | 107 (44) | 157 (64) |  | | Good, n (%) | 69 (29) | 45 (18) |  | | Poor, n (%) | 18 (7.4) | 15 (6.1) |  | | No image, n (%) | 48 (20) | 27 (11) |  | | Unknown, n (%) | 2 (0.008) | 0 (0) |  | | **Missing measurements** | | | | | LVEDD, n (%) | 34 (14) | 16 (6,5) | 0.007 | | RVEDD/LVEDD, n (%) | 40 (13) | 23 (9.5) | 0.02 | | Maximal IVC diameter, n (%) | 58 (24) | 38 (16) | < 0.001 | |
| --- | --- | --- | --- | --- | --- | --- | --- | --- | --- | --- | --- | --- | --- | --- | --- | --- | --- | --- | --- | --- | --- | --- | --- | --- | --- | --- | --- | --- | --- | --- | --- | --- | --- | --- | --- | --- | --- | --- | --- | --- | --- | --- | --- | --- | --- | --- | --- | --- | --- | --- | --- | --- | --- | --- | --- | --- | --- | --- | --- | --- | --- | --- | --- | --- | --- | --- | --- | --- | --- | --- | --- | --- | --- | --- | --- | --- | --- | --- | --- | --- | --- | --- | --- | --- | --- | --- | --- | --- | --- | --- | --- | --- | --- | --- | --- | --- | --- | --- | --- | --- | --- | --- | --- | --- | --- | --- | --- | --- | --- | --- | --- | --- | --- | --- | --- | --- | --- | --- | --- | --- | --- | --- | --- | --- | --- | --- | --- | --- | --- | --- | --- | --- | --- | --- | --- | --- | --- | --- | --- | --- | --- | --- | --- | --- |

Abbreviations: LVEDD, left ventricular end-diastolic diameter; RVEDD, right ventricular end-diastolic diameter; IVC, inferior vena cava.
