# Supplementary material for: Therapeutic impact of basic critical care echocardiography performed by residents after limited training
Source: Ann Intensive Care. 2024 Jul 29;14:119. doi: 10.1186/s13613-024-01354-7 (PMC11286607; doi:10.1186/s13613-024-01354-7)
Supplement: Supplementary file 3 — Supplementary Material 3. [file 13613_2024_1354_MOESM3_ESM.doc]

**Supplementary Table 2:** Answers to clinical questions provided by residents and experienced operators using basic critical care echocardiography

| Clinical questions | Number of clinical questions addressed by residents*^a^* | Number of clinical questions addressed by experienced operators*^a^* | *p* | Kappa coefficient*^b^* |
| --- | --- | --- | --- | --- |
| Global LV systolic function | 241/244(99) | 244/244(100) | 0.20 | 0.82 (0.75-0.90) |
| LV contractility*^c^* | 121/244(50) | 125/244(51) | 0.70 | 0.75 (0.63-0.87) |
| LV size | 238/244(98) | 243/244(99) | 0.12 | 0.78 (0.67-0.89) |
| RV dilatation | 231/244(95) | 238/244(97) | 0.10 | 0.74 (0.64-0.83) |
| RV dysfunction | 232/244(95) | 237/244(97) | 0.20 | 0.81 (0.68-0.94) |
| Pericardial effusion | 226/244(93) | 236/244(97) | 0.04 | 0.77 (0.63-0.92) |
| Tamponade | 226/244(93) | 236/244(97) | 0.04 | 1 (1-1) |
| IVC dilatation | 192/244(79) | 209/244(86) | 0.04 | 0.77 (0.65-0.88) |
| IVC inspiratory collapse*^d^* | 52/63(83) | 58/63(92) | 0.50 | 0.68 (0.48-0.90) |
| Massive mitral regurgitation | 220/244(90) | 226/244(93) | 0.30 | 0.80 (0.47-1) |
| Massive aortic regurgitation | 220/244(90) | 226/244(93) | 0,30 | 1 (1-1) |

^a^: Numbers in parentheses are percentages; *^b^*: 95% CIs are indicated in parentheses; *^c^*: indicate the ability to distinguish between a homogeneous and a heterogeneous left ventricular wall systolic thickening (only akinesia or dyskinesia were considered, as opposed to hypokinesia irrespective of its severity); *^d^*: in spontaneously breathing patients.

Abbreviations: LV, left ventricle; RV, right ventricle; IVC, inferior vena cava.
